# mTOR Signaling Regulates the Development and Therapeutic Efficacy of PMN-MDSCs in Acute GVHD

**DOI:** 10.3389/fcell.2021.741911

**Published:** 2021-12-23

**Authors:** Xiaoqing Li, Yixue Li, Qinru Yu, Lin Xu, Shan Fu, Cong Wei, Limengmeng Wang, Yi Luo, Jimin Shi, Pengxu Qian, He Huang, Yu Lin

**Affiliations:** ^1^ Bone Marrow Transplantation Center, The First Affiliated Hospital, School of Medicine, Zhejiang University, Hangzhou, China; ^2^ Liangzhu Laboratory, Zhejiang University Medical Center, Hangzhou, China; ^3^ Zhejiang Province Engineering Laboratory for Stem Cell and Immunity Therapy, Hangzhou, China; ^4^ Institute of Hematology, Zhejiang University, Hangzhou, China

**Keywords:** polymorphonuclear MDSCs, mTOR, acute GVHD, GVL effect, allogeneic HSCT

## Abstract

Myeloid-derived suppressor cells (MDSCs) represent a population of heterogeneous myeloid cells, which are characterized by their remarkable ability to suppress T cells and natural killer cells. MDSCs have been proven to play a positive role in protecting acute graft-versus-host disease (aGVHD). Here, we aimed to describe the mechanism behind how mTOR signaling regulates MDSCs’ generation and explore its prophylactic and therapeutic potential in aGVHD. Reducing mTOR expression retains myeloid cells with immature characteristics and promotes polymorphonuclear MDSC (PMN-MDSC) immunosuppressive function through STAT3-C/EBPβ pathway. Prophylactic transfusion of mTOR^KO^ PMN-MDSCs could alleviate aGVHD while maintaining the graft-versus-leukemia (GVL) effect, which could downregulate the Th1/Th2 ratio, decrease serum proinflammatory cytokines, and increase the proportion of regulatory T cells (Tregs) in aGVHD models at the early stage after transplantation. Moreover, transfusion therapy could promote the reconstruction and function of donor-derived PMN-MDSCs. Not only the percentage and the absolute number of donor-derived PMN-MDSCs significantly increased but also the immunosuppressive ability was much more robust compared to other groups. Altogether, these findings indicated that mTOR is an intrinsic regulator for PMN-MDSCs’ differentiation and immunosuppressive function. Together, mTOR^KO^ PMN-MDSC transfusion can play a protective role in alleviating cytokine storm at the initial stage and promoting the quantitative and functional recoveries of donor-derived PMN-MDSCs in aGVHD.

## Introduction

Allogeneic hematopoietic stem cell transplantation (allo-HSCT) is a potentially curative therapy for different hematopoietic diseases. The graft-versus-host disease (GVHD) represents one of the major complications and causes of death after allo-HSCT, where T cells play a central role in the pathogenesis and progression of acute GVHD (aGVHD) (Korngold and Sprent 1987; Vogelsang, Lee, and Bensen-Kennedy 2003). Recently, immune-regulatory cell therapy is gaining widespread attention in GVHD control, including mesenchymal stromal cells (MSCs) (Ringden et al., 2006; Le Blanc et al., 2008), regulatory T cells (Tregs) (Hess 2006; Koreth et al., 2011), and myeloid-derived suppressor cells (MDSCs) (Messmann et al., 2015; Zhang et al., 2019).

MDSCs represent a population of heterogeneous myeloid cells, precursors of dendritic cells (DCs), macrophages, and granulocytes, which accumulate in various pathophysiological settings, such as cancers, chronic inflammations, and autoimmune diseases (Gabrilovich and Nagaraj 2009). Currently, there is a lack of specific gene signatures or surface markers to identify MDSCs among myeloid cells due to their plasticity in different microenvironments. Therefore, their immunosuppressive ability become the most unique feature of MDSCs. In mice, MDSCs are historically defined as cells expressing both Gr1 and CD11b markers. The CD11b^+^Ly6G^−^Ly6C^hi^ subset is identified as monocytic MDSCs (M-MDSCs), while the CD11b^+^Ly6G^+^Ly6C^lo^ subgroup is defined as polymorphonuclear MDSCs (PMN-MDSCs) (Bronte et al., 2016). MDSCs exert their suppressive functions *via* several mechanisms including the expression of enzymes, such as arginase (Arg), nitric oxide synthase (NOS), and indamine 2,3-dioxygenase (IDO); releasing reactive oxygen species (ROS); and inducing Tregs and secreting cytokines (e.g., IL-6, IL-10, and TGF-β) (Dugast et al., 2008; Gabrilovich and Nagaraj 2009; Garcia et al., 2010; Deng et al., 2018).

It has been observed that a higher proportion of MDSCs in grafts was correlated to a lower incidence of aGVHD (Vendramin et al., 2014; Lv et al., 2015; Fan et al., 2017; Wang et al., 2019). The granulocyte colony-stimulating factor (G-CSF) is clinically used as a mobilizer to increase the number of circulating hematopoietic stem cells. Both PMN-MDSCs and M-MDSCs induced by G-CSF could exert suppressive function to reduce aGVHD (Perobelli et al., 2016; D'Aveni et al., 2015). After allo-HSCT, MDSCs recovering at the early stage after transplantation presented immunomodulatory properties to suppress donor-derived T cell proliferation and differentiation and promote Tregs development (Mougiakakos et al., 2013; Rieber et al., 2014; Guan et al., 2015). Besides, co-transfusing MDSCs generated *in vitro* could alleviate aGVHD, while preserving the graft-versus-leukemia (GVL) effect in murine models (Messmann et al., 2015; Zhang et al., 2019). Hence, a better understanding of how to enhance MDSCs’ suppressive ability and what are their regulatory networks *in vivo* requires to be further investigated.

Suppressive MDSCs can be induced and expanded by various methods (Zhou et al., 2010; Gomez et al., 2014). Culturing bone marrow cells for 4 days with G-CSF and granulocyte-macrophage (GM)-CSF can induce functional MDSCs able to inhibit allogeneic T cell responses *in vitro* and *in vivo* (Highfill et al., 2010). MDSCs induced by progenipoietin-1 (a synthetic G-CSF/Flt-3 ligand molecule) can promote transplant tolerance by inducing MHC class II-restricted, IL-10-secreting, antigen-specific Tregs (MacDonald et al., 2005). mTOR is an evolutionary conserved serine/threonine kinase that plays key regulatory roles in several biological processes, such as cell survival, proliferation, differentiation, metabolism, and autophagy (Beauchamp and Platanias 2013). The activity of mTOR affects the differentiation and functions of various innate and adaptive immune cells, including effector T cells, Tregs, and antigen-presenting cells (Delgoffe et al., 2009; Thomson et al., 2009; Rao et al., 2010). Targeting mTOR on the recruitment, expansion, and function of MDSCs has been reported in different disease models. Rapamycin, an mTOR inhibitor, regulated the differentiation and suppressive function of MDSCs in protecting against immunological hepatic injury and aGVHD (Zhang et al., 2014; Lin et al., 2018). Additionally, rapamycin could prolong cardiac or corneal allograft survival by inducing MDSCs after transplantation (Nakamura et al., 2015; Wei et al., 2018). However, the underlying mechanisms of how mTOR signals regulate MDSCs are currently not well defined.

In our present study, we demonstrated that the mTOR signaling regulated the suppressive function of PMN-MDSCs through the STAT3-C/EBPβ pathway. Prophylactic transfusion of mTOR-deficient PMN-MDSCs could play a protective role in alleviating cytokine storm at the initial stage and promote a quantitative and functional recovery of donor-derived PMN-MDSCs in aGVHD models. We also explored the mechanism of how mTOR regulated MDSCs and demonstrated that mTOR-deficient PMN-MDSCs might become an effective approach for aGVHD prevention in allo-HSCT.

## Materials and Methods

### Mice

Experiments were performed with age-matched mTOR^fl/fl^ (referred to as wild-type, WT control) mice and LysM-Cre mTOR^fl/fl^ (referred as to mTOR knockout, mTOR KO) mice. LysM-Cre mTOR^fl/fl^ mice were crossed with LysM-Cre and mTOR^fl/fl^ mice, confirmed by genotyping, which were kindly provided by Prof. H Shen. BALB/c (H-2Kd) and C57BL/6 (H-2Kb) mice were directly purchased from SLAC Laboratory Supplies (China). The CD45.1 congenic mice (C57BL/6) were a gift from Prof. P Qian. Animal experiments were conducted under specific pathogen-free conditions in the Laboratory Animal Center of Zhejiang University and approved by the Institutional Animal Care and Use Committee of Zhejiang University.

### Reagents and Antibodies

The antibodies used for flow cytometric analyses are listed in Supplementary Table S1. Purified Fc Block (anti-mouse FcRγII/III monoclonal antibody) and isotype control antibodies were obtained from eBioscience. Rapamycin and STAT3 inhibitor were purchased from TargetMol (China). Carboxyfluorescein diacetate succinimidyl ester (CFSE) was purchased from Invitrogen (United States). L-NG-Monomethylarginine, Acetate Salt (L-NMMA) and N(omega)-hydroxy-nor-L-arginine (nor-NOHA) were purchased from MCE (United States). Recombinant murine IL-2 was obtained from PeproTech (United States).

### aGVHD and GVL Models

The MHC-mismatched aGVHD model was used as previously established (Hill et al., 1997; Lin et al., 2018). Briefly, BALB/c mice received 5 × 10^6^ bone marrow (BM) cells (Mouse CD3 Positive Selection, Biolegend) after lethal irradiation with 8 Gy (split doses of 2 × 4.0 Gy 4 h apart) for the C57BL/6 → BALB/c combinations. To induce aGVHD, CD3^+^ T cells were isolated (Pan T Cell Isolation Kit, Miltenyi Biotec) from donor’s spleens and given at a dosage of 5 × 10^5^ i.v. on day 0. Clinical and histopathological scores were assigned following the criteria described by Hill et al. (1997). In GVL models, recipients received grafts containing 2 × 10^4^ A20 tumor cells. Representative samples of GVHD target organs were excised from recipients on day 21 post-BMT and subjected to pathology scoring as previously described (Lin et al., 2018).

### Cellular Flow Cytometry

First, cells were stained with surface markers for 30 min at 4°C in stain buffer (BD Biosciences) and washed with PBS (2% FBS). Foxp3 and granzyme B analyses were performed following the manufacturer’s instructions (True-Nuclear Transcription Factor Buffer Set, BioLegend). For intracellular staining, cells were fixed and permeabilized with Cytofix/Permwash buffer (BD Biosciences) for 30 min at 4°C. Then, they were incubated with intracellular antibodies for another 30 min. Isotype controls were used for intracellular staining, whereas extracellular markers were gated according to the fluorescence minus one control. For T cell intracellular staining, cells isolated from each group were individually cultured for 6 h in the presence or absence of PMA (20 ng/ ml) and ionomycin (1 μg/ ml), with GolgiStop brefeldin A solution (BD Biosciences) during the last 4 h. The FlowJo v.10 software (BD Biosciences) was used to analyze and calculate the MFI for each sample.

### Transduction of shRNA

The specific STAT3 and control shRNA were synthesized by Shanghai GenePharma Co., Ltd (Ling and Arlinghaus 2005). The control (E20150630A) and specific C/EBPβ (V3LMM_504726) shRNA were purchased from Dharmacon. Briefly, target cells were transduced with lentiviruses accompanied with polybrene 8 μg/ ml and centrifuged at 500*g* for 1 h. After 12 h, the serum-free culture medium was replaced by a fresh complete medium and incubated for 48 h. The transfection efficiency was analyzed by flow cytometry.

### Cell Isolation and Culture

A20 (BALB/c B-cell lymphoma cell line) was bought from ATCC and cultured in RPMI-1640 (Gibco) supplemented with 10% FBS. To detect immunosuppressive function, PMN-MDSCs and M-MDSCs were sorted by MDSC Isolation Kit (Miltenyi Biotec) or flow cytometry cell sorting (BD FACSAria II). Human CD15^+^ cells were positively isolated by human CD15 MicroBeads (Miltenyi Biotec) from the bone marrow of healthy volunteers. All subjects signed informed consent forms. The purity was normally over 95% as assessed by flow cytometry (CytoFLEX LX, Beckman). The isolated PMN-MDSCs were cultured in RPMI-1640 (Gibco) with 2 mM l-glutamine, 10 mM HEPES, 1 mM sodium pyruvate, 50 mM 2-mercaptoethanol, 1% penicillin/streptomycin (Sigma), and 10% fetal bovine serum (Gibco) at 5% CO_2_ and 37°C. Media were supplemented with 20 ng/mL GM-CSF. PMN-MDSCs co-cultured with T cells activated by anti-CD3/CD28 antibodies (Invitrogen) for 3 days were harvested for further studies.

### Quantitative RT-PCR

RNA was extracted using TRIzol (Invitrogen), and cDNA was synthesized using Takara. A SYBR Premix Ex Taq (Takara) Real-Time PCR system was used for quantitative PCR. The primers used are shown in Supplementary Table S2. The expression of target genes was determined using the comparative CT (ΔΔCT) method and presented as the “fold change” relative to control samples.

### Functional Assay of PMN-MDSCs

The *in vitro*-suppressive function of MDSCs was assessed by determining their ability to inhibit T cell activation as described previously. Briefly, purified T cells from the spleen were labeled with CFSE (2.5 mM) and plated at a density of 1 × 10^5^ cells/well in 5 μg/ml anti-CD3 antibody-coated, round-bottom, 96-well plates (4°C, overnight) and in the presence of 2 μg/ml soluble anti-CD28 antibody. Isolated MDSCs were added to the wells at the same size ratio. Cell proliferation was determined 3.5 days later by CFSE expression.

### Cytokine Measurements

Serum samples were obtained from different recipients at the time specified. G-Series Mouse Inflammation Array (Ray Biotech) was used for semi-quantitative measurement of mouse cytokines from serum according to the manufacturer’s instructions.

### Western Blot Analyses

Protein was extracted from cells using RIPA buffer (Beyotime Biotechnology) with protease and phosphatase inhibitors (Roche Diagnostics GmbH). Samples were electrophoresed through 10% polyacrylamide gels and immunoblotted with the relevant antibodies using standard methods. The antibodies for β-actin (4970), mTOR (2972), STAT3 (9139), p-STAT3 (9145), and C/EBPβ (3087) were purchased from CST.

### RNA Sequencing

Total RNA was isolated using TRIzol (Invitrogen), and sequencing libraries were prepared from 10 to 100 ng total RNA using the TruSeq RNA Sample Preparation Kit v2 (Illumina). RNA sequencing (RNA-seq) and the subsequent analyses were completed by Huada Gene Biological Company (China). The heatmap was constructed using the “heatmap” package in R 3.4.4. The RNA sequencing data were deposited in the Sequence Read Archive (SRA) database: PRJNA726285.

### Statistical Analyses

Experiments were performed with at least three independent biological replicates, and data are presented as means ± standard deviations (SD). Statistical analyses were performed by conducting an unpaired (two-sided) Student’s *t*-test for normally distributed data. The Mann–Whitney *U* (MWU) test was conducted if the data failed to meet normality criteria. Comparison of the survival curves was generated using the Kaplan–Meier method and compared by log-rank test. To obtain unbiased data, the histopathologic scoring of the aGVHD severity was performed by researchers who were blinded to the treatment groups. A *p* < 0.05 was considered statistically significant. All statistical analyses and graphics generation were performed using GraphPad Prism v.7.0 (GraphPad Software, United States).

## Results

### Reducing mTOR Expression Retains Myeloid Cells’ Immature Characteristics and Promotes PMN-MDSCs’ Immunosuppressive Function

**FIGURE 1:**
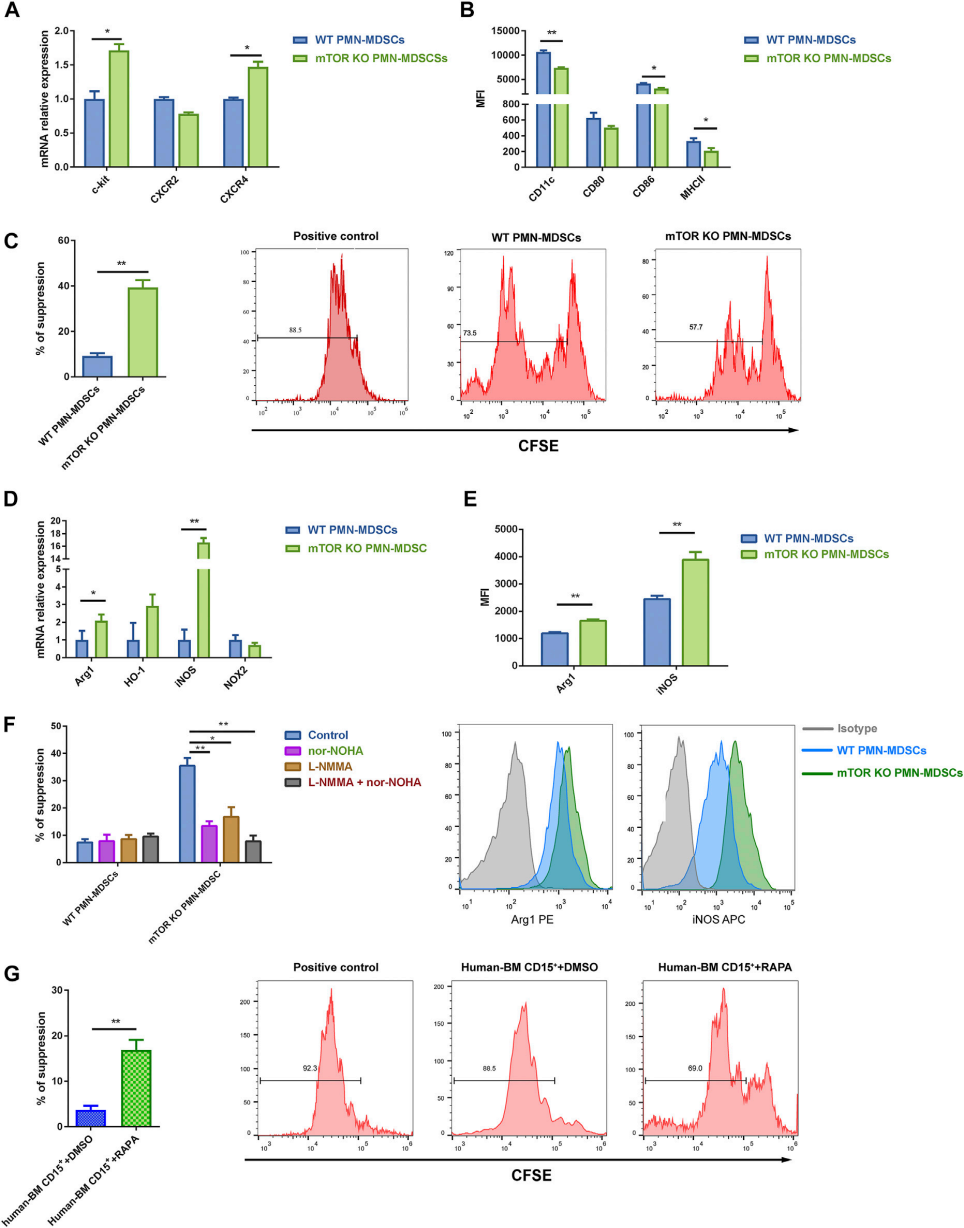
mTOR depletion induced the suppressive function of bone marrow (BM)-derived polymorphonuclear myeloid-derived suppressor cells (PMN-MDSCs). **(A)** PMN-MDSCs were generated *in vitro* from mTOR^fl/fl^ and LysM-Cre mTOR^fl/fl^ mice BM cells co-cultured with active T cells for 3 days. High transcriptional levels of neutrophil precursor markers were detected in mTOR^KO^ PMN-MDSCs. **(B)** mTOR depletion downregulated surface maturation markers. **(C)** Carboxyfluorescein diacetate succinimidyl ester (CFSE)-labeled splenic T cells were stimulated with anti-CD3/CD28 in the presence of wild-type (WT) or mTOR^KO^ PMN-MDSCs at a 1:1 ratio for 3.5 days. The suppression of splenic T cell proliferation was analyzed and calculated as PMN-MDSCs’ suppressive ability (mean ± SD; *n* = 8). **(D)** Transcript levels of the MDSC immunosuppressive markers were determined by qPCR (mean ± SEM; *n* = 5). **(E)** Flow cytometry analyses of Arg1 and iNOS expression in WT and mTOR^KO^ PMN-MDSCs after co-culturing with active T cells. Representative Arg1 and iNOS histogram showing overlays of WT PMN-MDSCs, mTOR^KO^ PMN-MDSCs, and the appropriate isotype control. **(F)** Arg1 inhibitor (nor-NOHA) and/or iNOS inhibitor [L-NG-Monomethylarginine, Acetate Salt (L-NMMA)] were added in PMN-MDSCs’ suppressive function assays. **(G)** CD15^+^ BM cells isolated from healthy donors were pretreated with rapamycin for 4 h, which significantly suppressed the active peripheral blood mononuclear cell (PBMC) proliferation compared to DMSO treatment (control). Data are shown as mean ± SD of one representative experiment of three–four experiments performed. **p < 0.01, *p < 0.05; *p*-values reflected analyses with two-tailed unpaired Student’s *t*-test.

As we previously demonstrated, the mTOR inhibitor rapamycin could induce a strong immunosuppressive function in PMN-MDSCs from murine BM *in vitro*, which prompted us to explore how mTOR regulates MDSCs’ generation and function. Thus, we generated mice with myeloid-specific mTOR deficiency by crossing mTOR^fl/fl^ with LysM-Cre recombinase mice. LysM-Cre mTOR^fl/fl^ (referred to as mTOR^KO^) mice presented a similar absolute number of BM and spleen (SP) cells with mTOR^fl/fl^ (referred to as WT) control mice (Supplementary Figure S1A). The proportion of PMN-MDSCs and M-MDSCs in the BM and SP was comparable in WT and mTOR^KO^ mice (Supplementary Figure S1B). mTOR knockout increased the percentage of proliferating cells in S and G2/M phases and elevated resistance to apoptosis under active T cell stimulation (Supplementary Figures S1C, D). The expression of c-Kit and CXCR4 was higher in mTOR^KO^ PMN-MDSCs (Figure 1A), which revealed that they presented precursor myeloid cells characteristics. Additionally, mTOR^KO^ PMN-MDSCs had lower expression of MHC II and CD86 compared to WT PMN-MDSCs (Figure 1B), suggesting that loss of mTOR maintains PMN-MDSCs in an immature state.

Next, we determined the effect of mTOR deficiency on the suppressive function of PMN-MDSCs. T cell proliferation assays were performed by co-incubating BM-derived PMN-MDSCs with splenic T cells. The mTOR^KO^ PMN-MDSCs expressed a stronger immunosuppressive ability compared with WT PMN-MDSCs (Figure 1C), while mTOR deficiency ameliorated M-MDSCs’ immunosuppressive ability (Supplementary Figure S2A). Due to the larger amount of PMN-MDSCs compared to M-MDSCs, focusing on PMN-MDSCs as an overall treatment strategy deserves further investigation. Then, we explored the molecular mechanisms underlying their suppressive function. Arg1 and iNOS were significantly upregulated at transcriptional and protein levels in mTOR^KO^ PMN-MDSCs (Figure 1D, E). The administration of L-NMMA (an iNOS inhibitor) or nor-NOHA (an Arg1 inhibitor), either alone or combined, efficiently reduced immunosuppressive activity (Figure 1F).

Next, we hypothesized whether mTOR played the same role in human myeloid cells. After treating healthy donor BM cells with rapamycin for 4 h, the number of CD15^+^ cells and PMN-MDSC and M-MDSC proportions were not affected (Supplementary Figure S2B). The rapamycin pretreatment induced human BM CD15^+^ cells’ immunosuppressive function, but the variation degree was much smaller than that of murine cells (Figure 1G). These results showed that mTOR inhibition could promote PMN-MDSCs’ suppressive function both in mice and human BM cells.

### Reducing mTOR Expression Enhances STAT3 Activity in PMN-MDSCs

**FIGURE 2:**
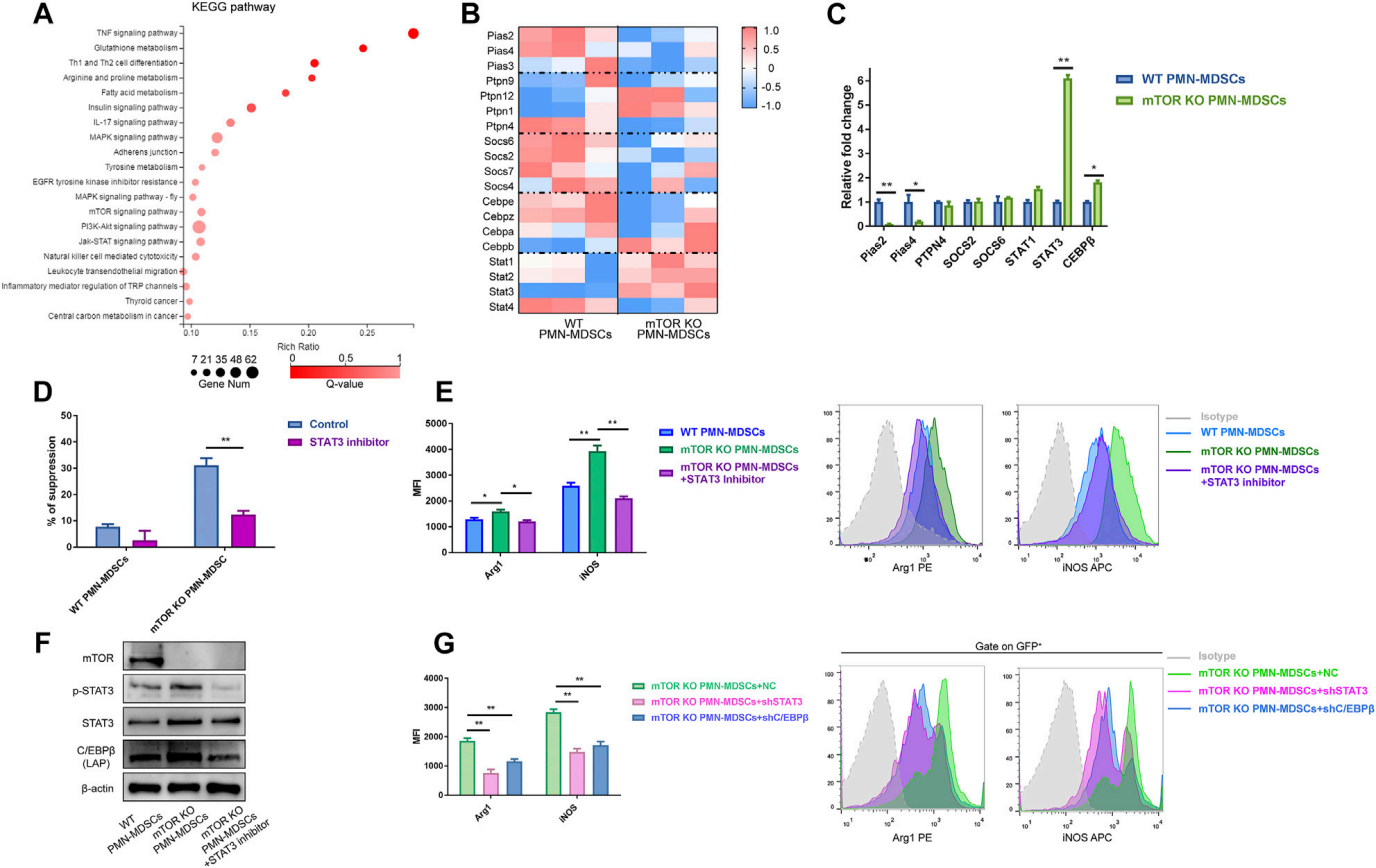
Reducing mTOR expression mainly regulated PMN-MDSCs through the STAT3-C/EBPβ pathway. **(A)** Kyoto Encyclopedia of Genes and Genomes (KEGG) pathway enrichment analysis of differentially expressed genes between WT and mTOR^KO^ PMN-MDSCs. **(B)** Heatmap showing different expressions of STAT3-related genes. **(C)** qPCR validations of selected genes (mean ± SEM, *n* = 5). **(D)** mTOR^KO^ PMN-MDSCs were generated with or without the STAT3 inhibitor Stattic (10 µM) for 4 h followed by co-culture with activated splenic T cells for 3.5 days at 1:1 ratios (mean ± SD, *n* = 6). **(E)** Effects of Stattic on Arg1 and iNOS expressions of mTOR^KO^ PMN-MDSCs. The representative histogram shows different expressions of suppressive markers among groups. **(F)** STAT3-C/EBPβ pathway western blot analyses. **(G)** The decline of Arg1 and iNOS expressions in mTOR^KO^ PMN-MDSCs after transfection with STAT3-shRNA or C/EBPβ-shRNA. Representative histogram of five experiments. **p < 0.01, *p < 0.05; *p*-values reflected analyses with the Mann–Whitney *U* (MWU) test.

Furthermore, we performed RNA-seq to uncover the molecular mechanisms underlying how mTOR deficiency rejuvenated PMN-MDSCs. A total of 2,860 differentially expressed genes were identified, 631 upregulated and 2,175 downregulated (Supplementary Figure S3A). Particularly, NOS2 showed a pronounced change in mTOR^KO^ PMN-MDSCs, which provided another evidence for the suppressive ability elevation (Supplementary Figure S3B). Through Kyoto Encyclopedia of Genes and Genomes (KEGG) analysis, many immune-related pathways were identified including TNF signaling, PI3K-Akt, and JAK-STAT (Figure 2A). STAT3 and C/EBPβ were enriched in multiple signaling pathways. Several negative regulators of STAT3 signaling including Pias, SOCS, and PTP cluster were downregulated in mTOR^KO^ PMN-MDSCs, but not in WT PMN-MDSCs (Figure 2B). The qPCR results validated significant decreases in Pias2 and Pias4 in mTOR^KO^ PMN-MDSCs; moreover, the upregulation of STAT3 and C/EBPβ was also demonstrated (Figure 2C). We verified the role of STAT3 in suppressive function using the STAT3 inhibitor, Stattic. We found that Stattic could significantly reduce the mTOR^KO^ PMN-MDSCs’ suppressive function (Figure 2D). Meanwhile, the expression of Arg1 and iNOS was downregulated (Figure 2E). Previous studies have shown that C/EBPβ is a downstream target of mTOR and STAT3 signaling pathways (Bitto et al., 2021). Western blot analyses confirmed that C/EBPβ (at 35 kDa) increased in mTOR^KO^ PMN-MDSCs and concurrently decreased with the downregulated STAT3 phosphorylation in the group treated with Stattic (Figure 2F). This indicated that STAT3 activation was required for C/EBPβ expression. To further verify the relationship between STAT3-C/EBPβ signaling and the mTOR^KO^ PMN-MDSC suppressive function, shRNA was performed to knockdown STAT3 and C/EBPβ in mTOR^KO^ PMN-MDSCs, respectively. STAT3 activation was not affected by C/EBPβ-shRNA (Supplementary Figure S4A, B), but the expression of Arg1 and iNOS decreased in both STAT3-shRNA and C/EBPβ-shRNA groups (Figure 2G). These results indicated that mTOR regulated the suppressive function of PMN-MDSCs through STAT3-C/EBPβ pathway activation.

### mTOR^KO^ PMN-MDSCs Could Alleviate aGVHD While Retaining the GVL Effect

**FIGURE 3:**
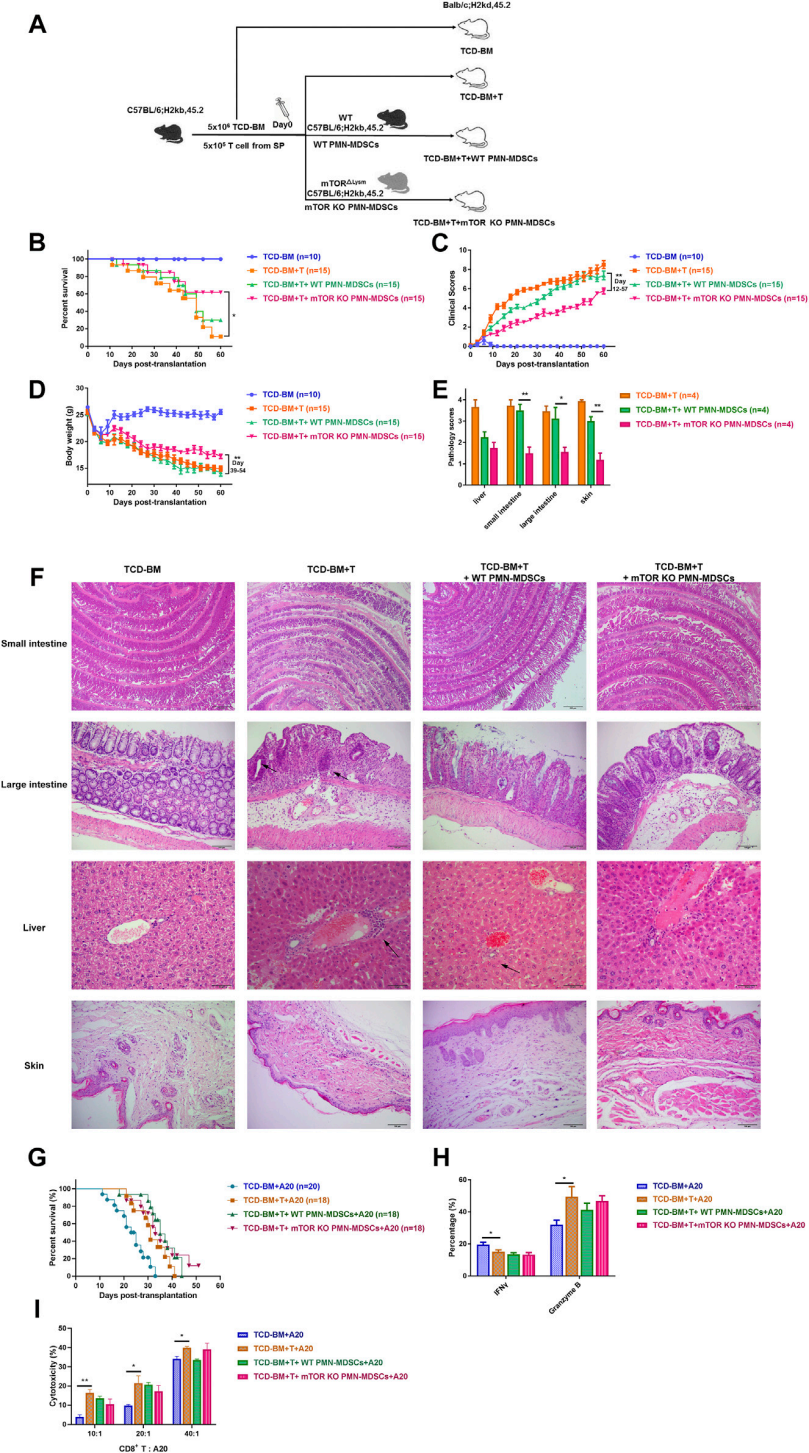
Transferring mTOR^KO^ PMN-MDSCs could alleviate acute graft-versus-host disease (aGVHD) while retaining the graft-versus-leukemia (GVL) effect. **(A)** Schematic diagram of the experimental design. BALB/c mice received allo-genetic C57B/L6 grafts in the absence or presence of 1 × 10^6^ WT or mTOR^KO^ PMN-MDSCs after lethal irradiation. **(B)** The improvements were observed in survival time (log-rank test, *p* < 0.05). **(C)** aGVHD clinical scores (days 12–57, *p* < 0.01) and **(D)** weight restoration (days 3–27, *p* < 0.05; days 39–54, *p* < 0.01) in the TCD-BM + T cells + mTOR^KO^ PMN-MDSC group. **(E)** Histopathological scores of aGVHD target tissues on day 21 after allogeneic hematopoietic stem cell transplantation (allo-HSCT). **(F)** Representative sections of organs are shown for each group. The area indicated by the arrow shows infiltrated inflammatory cells. **(G**) The survival of GVL models induced by A20 leukemic cell (H-2d) injection into recipients. **(H)** The frequency of donor-derived interferon-γ (IFN-γ) production and granzyme B expression in CD8^+^ splenic T cells on day 14. **(I)** The cytotoxic activity of donor CD8^+^ T cells was analyzed on day 14 after allo-HSCT. The data above are presented as mean ± SD and was assessed by unpaired Student’s *t*-test (two-sided) or MWU test, pooled from two–three independent experiments, with at least five mice per group. Numerals in brackets indicate the number of mice tested.

Since mTOR^KO^ PMN-MDSCs displayed a remarkable immunosuppressive function, we further explored their preventive and therapeutic capacity in murine aGVHD models. First, lethally irradiated BALB/c mice (H-2Kd) were reconstituted with T cell-depleted (TCD)-BM alone or TCD-BM plus CD3^+^ splenic T cells from B6 mice (H-2Kb). Then, WT PMN-MDSCs or mTOR^KO^ PMN-MDSCs were transfused with TCD-BM + T cells (Figure 3A). Mice who received TCD-BM alone achieved long-term survival within 60 days. Mice in the TCD-BM + T cells group had poor survival with a mortality rate of around 90% at 60 days and presented severe clinical aGVHD symptoms, such as weight loss, hunched posture, hair loss, and diarrhea. Histological examination of the intestine, liver, and skin showed severe tissue damage and lymphocyte infiltration. Infusion of WT PMN-MDSCs did not effectively improve the survival or alleviate the aGVHD symptoms. However, in the mTOR^KO^ PMN-MDSC infusion group, lower mortality rate and aGVHD scores were observed, and nearly 60% of the mice survived more than 60 days (Figure 3B–D). Lymphocyte infiltration and tissue damage were remarkably relieved, and pathological scores of the liver, intestine, and skin decreased in the mTOR^KO^ PMN-MDSC infusion group (Figure 3E, F). These observations suggested that mTOR^KO^ PMN-MDSC co-transfusion was an effective method to prevent aGVHD after allo-HSCT. However, when the mTOR^KO^ PMN-MDSC transfusion was performed on day 14 after aGVHD onset, the therapeutic ability was limited (Supplementary Figure S5A). The survival and aGVHD clinical and pathological scores did not differ among the TCD-BM + T cells, WT PMN-MDSC transfusion, and mTOR^KO^ PMN-MDSC transfusion groups (Supplementary Figure S5B–F).

Then, A20 cells were injected into recipients to induce tumor development to clarify the influence of mTOR^KO^ PMN-MDSCs on the GVL effect. Mice that received only TCD-BM plus A20 cells died within 32 days due to leukemia progression. Mice in the TCD-BM + T cells plus A20 cells group displayed minimal tumor infiltration but died due to aGVHD-related damage. The mTOR^KO^ PMN-MDSC transfusion group showed the best survival compared with other groups (Figure 3G). These results indicated that mTOR^KO^ PMN-MDSCs could actively suppress aGVHD after allo-HSCT while retaining GVL activity. To explore the underlying mechanisms, we examined the activation and functional activity of donor-derived CD8^+^ T cells, known as the main effector cells governing anti-tumor response. Intercellular staining of effective markers revealed no difference in interferon-γ (IFN-γ) or granzyme B expression in CD8^+^ T cells among the mTOR^KO^ PMN-MDSC treatment, WT PMN-MDSC treatment, and untreated groups (Figure 3H). Correspondingly, isolated CD8^+^ T cells from mTOR^KO^ PMN-MDSC-treated mice exhibited a cytotoxic activity against allogeneic A20 cells, which showed no clear difference compared with other groups (Figure 3I). These data provided further evidence that the cytotoxic capabilities of donor-derived CD8^+^ T cells were unaffected by mTOR^KO^ PMN-MDSCs.

### mTOR^KO^ PMN-MDSCs Could Alleviate Cytokine Storm at aGVHD Initial Stage

**FIGURE 4:**
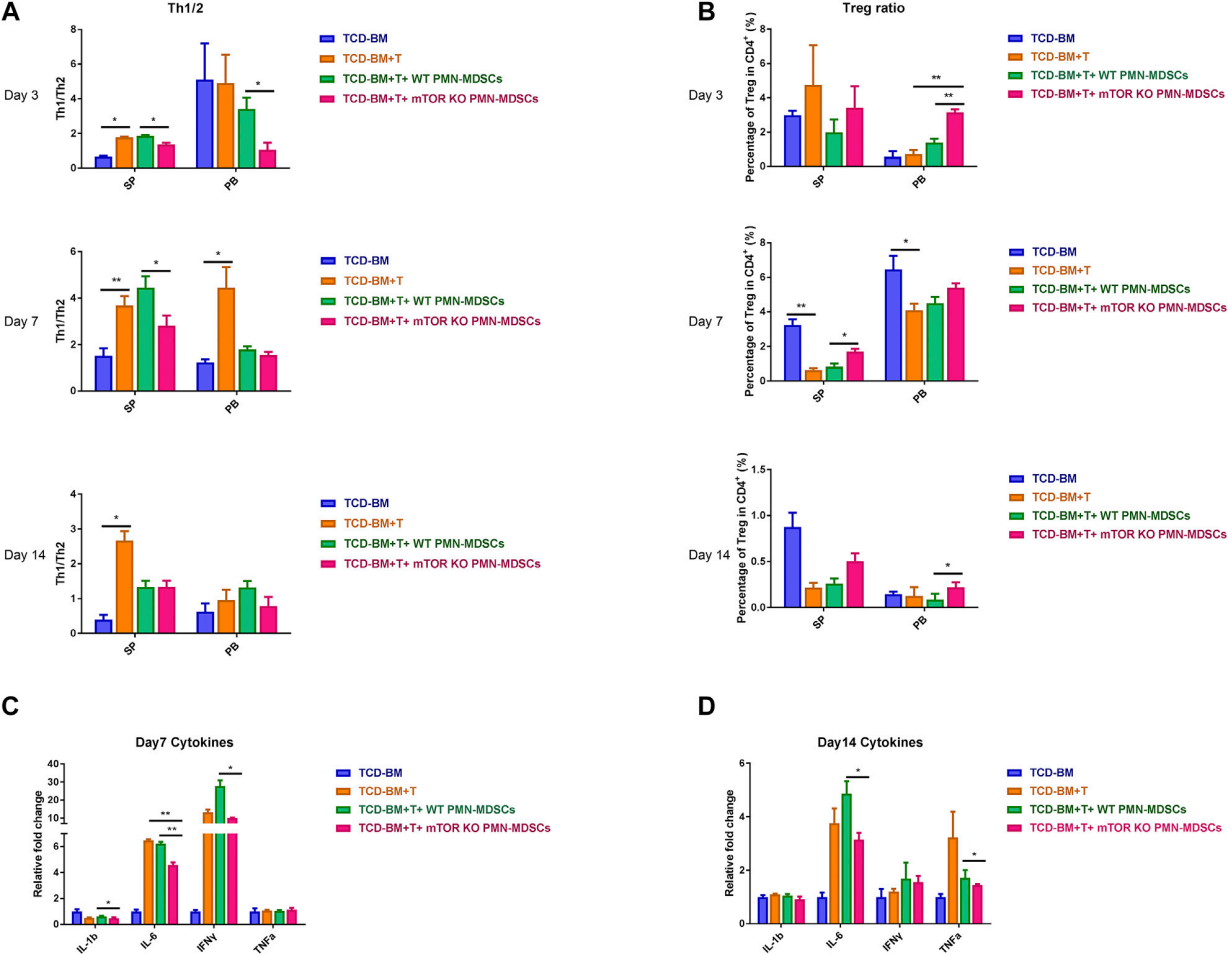
mTOR^KO^ PMN-MDSCs alleviated cytokine storm in aGVHD mice and restrained Th1/Th2 cell differentiation but promoted Tregs induction. **(A–B)** Th1/Th2 ratios **(A)** and Tregs percentage out of the CD4^+^ T cells total **(B)** detected in the spleen (SP) and peripheral blood (PB) on days 3, 7, and 14 after allo-HSCT. Data are presented as mean ± SD (*n* = 6). One representative experiment of three is shown. **(C–D)** The relative level of inflammatory cytokines in serum was measured on days 7 **(C)** and 14 **(D)**. Data are presented as mean ± SD (*n* = 4). The *p-*values were determined by MWU test, *p < 0.05, **p < 0.01. One representative experiment of three is shown.

Our previous findings confirmed that only co-transfusing mTOR^KO^ PMN-MDSCs with grafts could exert a protective effect of aGVHD development, rather than a therapeutic activity after aGVHD occurrence. Therefore, we speculated that the infused mTOR^KO^ PMN-MDSCs could restrain donor-derived T lymphocyte activation and expansion at a very early stage of aGVHD. Thus, we analyzed the proportions of several T cell subsets in each group on days 3, 7, and 14 after transplantation. In peripheral blood (PB) and SP, the T helper cell type 1 versus type 2 (Th1/Th2) ratio was remarkably elevated in TCD-BM + T cells and WT PMN-MDSC infusion groups, whereas decreased in the mTOR^KO^ PMN-MDSCs infusion group (Figure 4A). Since inducing Tregs generation is considered as an important MDSC mechanism to suppress GVHD progression (Edinger et al., 2003; Zhao et al., 2008), we further detected the percentage of Tregs in total CD4^+^ T cells from PB and SP. During the early period after transplantation, mice who received mTOR^KO^ PMN-MDSCs had a higher percentage of Tregs compared to other aGVHD groups (Figure 4B). Proinflammatory cytokines in serum including IL-1β, IL-6, TNF-a, and IFN-γ remarkably increased in the TCD-BM + T cells and WT PMN-MDSC infusion groups, but reduced in the mTOR^KO^ PMN-MDSC infusion group on days 7 and 14 (Figure 4C, D). These findings demonstrated that adoptive transferring of mTOR^KO^ PMN-MDSC could suppress the activation and proliferation of allo-active T cells at the beginning of aGVHD, thereby stopping cytokine storm amplification.

### mTOR^KO^ PMN-MDSCs Could Promote a Quantitative and Functional Recovery of Donor-Derived PMN-MDSCs at aGVHD Later Stage

**FIGURE 5:**
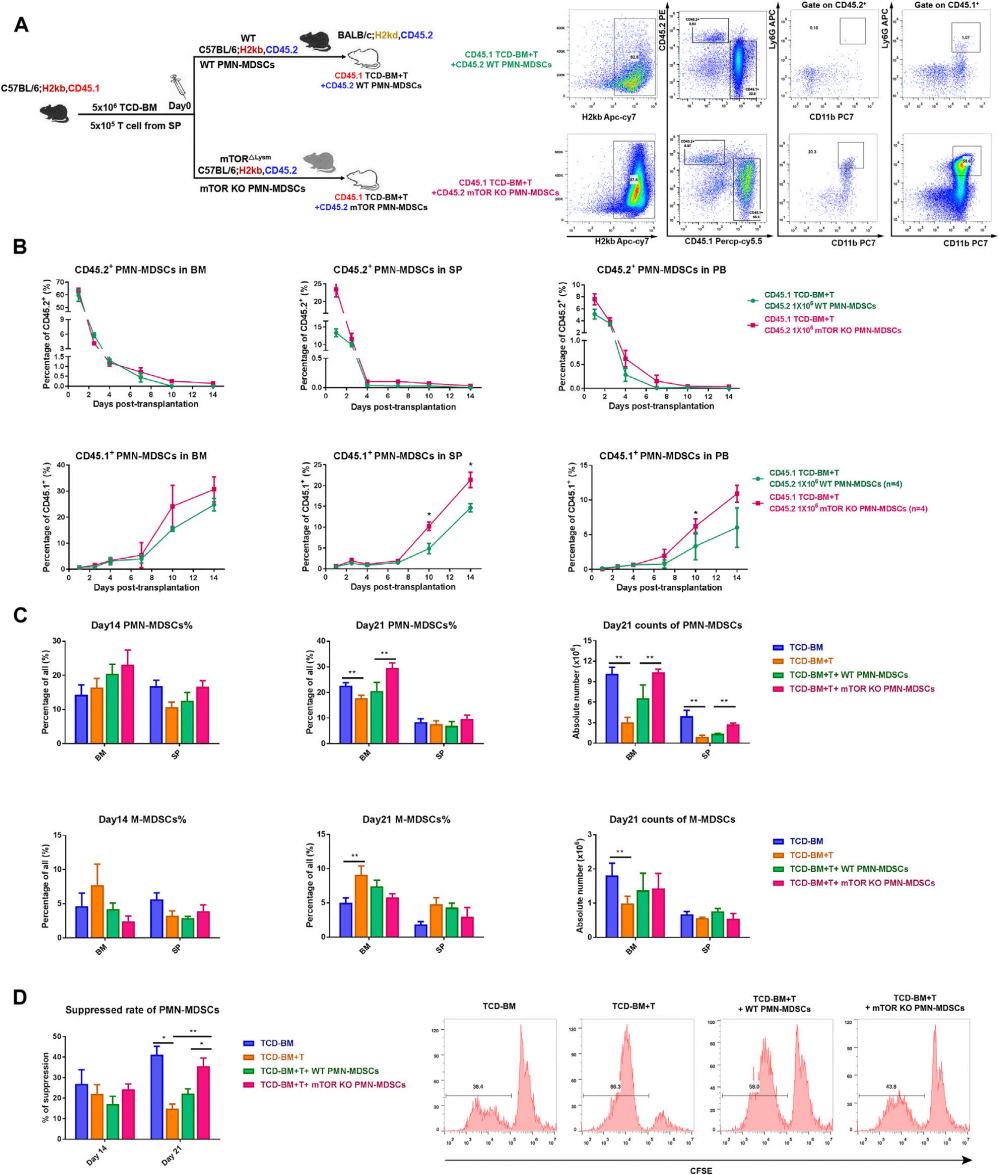
Transfused mTOR^KO^ PMN-MDSCs exerted suppressive effects during the early post-transplant period and contributed to donor-derived PMN-MDSC functional recovery. **(A)** Lethally irradiated CD45.2 BALB/c recipients reconstituted with CD45.1 congenic C57BL/6 mice **(left)**. Gating strategy to discriminate the original donor (CD45.1) or host (CD45.2) PMN-MDSCs in the BM, SP, and PB, and the flow analyses of BM on day 10 was used as an example **(right)**. **(B)** Transferred CD45.2^+^ (top row) and donor-derived CD45.1^+^ (bottom row) PMN-MDSCs were monitored on days 1, 3, 5, 7, 10, and 14 in the BM, SP, and PB. **(C)** PMN-MDSC and monocytic MDSC (M-MDSC) percentage and absolute number in the BM and SP. **(D)** The suppressive ability of BM PMN-MDSCs in each group on days 14 and 21. Data are presented as mean ± SD (*n* > 3) of one representative experiment out of three. The *p*-values were determined by unpaired Student’s *t*-test (two-sided) or MWU test, *p < 0.05, **p < 0.01.

Next, we investigated how long the infused mTOR^KO^ PMN-MDSC could persist *in vivo* and whether they could play a long-term suppressive function. Thus, BALB/c mice (H2Kd, CD45.2) received B6-derived TCD-BM + T cells (H2Kb, CD45.1) with WT or mTOR^KO^ PMN-MDSCs (H2kb, CD45.2) to distinguish the host-derived or co-transfused PMN-MDSCs by CD45.1 and CD45.2 markers (Figure 5A). Both the transfused CD45.2^+^ WT and mTOR^KO^ PMN-MDSCs remained at a relatively high level on day 1 after transplantation, then markedly declined over time. After the transfusion, we found that either WT or mTOR^KO^ PMN-MDSCs (CD45.2, H-2Kb) persisted for no more than 10 days in BM, SP, and PB. In contrast, donor-derived PMN-MDSCs (CD45.1, H-2Kb) rapidly differentiated from progenitor cells, and the difference between the two groups gradually increased over time (Figure 5B). Therefore, these results indicated that mTOR^KO^ PMN-MDSC transfusion could promote a better recovery of donor-derived PMN-MDSCs.

Then, we measured donor-derived PMN-MDSCs and M-MDSCs in BM and SP on days 14 and 21 after transplantation. The percentage and absolute number of PMN-MDSCs remarkably increased in BM and SP of the mTOR^KO^ PMN-MDSC-infused group (Figure 5C). The immunosuppressive ability of PMN-MDSCs from the mTOR^KO^ PMN-MDSC infusion group was also much more robust on day 21 compared to other groups (Figure 5D). Meanwhile, M-MDSCs possessed a powerful suppressive function but with no significant difference among groups (Supplementary Figure S6A). Overall, our results showed that although transfused mTOR^KO^ PMN-MDSCs could not maintain a long-lasting persistence in BM, SP, and PB, they could promote the proliferation and functional recovery of donor-derived PMN-MDSCs.

## Discussion

The sensor mTOR is important for various environmental or cellular changes, such as nutrient deprivation, energy insufficiency, and cytokine stimulation. Also, mTOR is important to control the fate of diverse immune cells (Powell et al., 2012; Yang et al., 2014). Generally, cytokines such as G-CSF, GM-CSF, IL-1β, IL-6, IL-4, and S100A8/A9 are essential to induce immunosuppressive function of BM-derived PMN-MDSCs (Talmadge and Gabrilovich 2013). In the current study, we found that isolated mTOR^KO^ PMN-MDSCs could directly exhibit a strong suppressive ability without long-term and complex cytokine induction *in vitro*. Consistent with previous studies, our results showed that mTOR inhibition could decrease M-MDSCs’ immunosuppressive ability (Wu et al., 2016), which revealed that the mTOR signaling might have an opposite regulatory function on these two subpopulations. Thus, we investigated how mTOR regulates PMN-MDSCs’ generation and function. According to Evrard et al. (2018), neutrophils in the BM can be identified by mass cytometry and cell cycle-based analyses as neutrophil precursors, immature neutrophils, and mature neutrophils. Among them, immature neutrophils have suppressive ability. Correspondingly, we found that the proportion of the immature subset increased in the BM of mTOR^KO^ mice (Supplementary Figure S7). These findings implied that the mTOR deficiency maintained myeloid cells in an immature state, which might be consistent with its functional properties.

The JAK-STAT signaling is considered to play a critical role in the expansion and function of MDSCs (Vasquez-Dunddel et al., 2013; Waldmann and Chen 2017). STAT3 is an essential transcription factor that activates genes involved in the differentiation and proliferation of MDSCs, such as c-myc, cyclin D1, and S100A8/A9, and induces genes associated with suppressive functions including NADPH oxidase subunits, p47^phox^ and gp91^phox^, and C/EBPβ (Marigo et al., 2010). C/EBPβ is an intronless gene that generates three isoforms—liver activating protein* (LAP*, 38 kDa), liver activating protein (LAP, 35 kDa), and liver inhibitory protein (LIP, 20 kDa)—which are highly expressed in innate immune cells (Guo, Li, and Tang 2015). When mTORC1 activity is low (e.g., with the presence of rapamycin), the downstream translation of C/EBPβ-LAP is favored (Bitto et al., 2021). We observed an increased level of C/EBPβ-LAP in mTOR^KO^ PMN-MDSCs, which was STAT3 signal-dependent. The suppressive function of mTOR^KO^ PMN-MDSCs could be abolished when STAT3 activation or C/EBPβ-LAP expression was obstructed. Therefore, we concluded that mTOR regulated the suppressive function of PMN-MDSCs *via* STAT3-C/EBPβ pathway activation. Recent studies have demonstrated that mTOR and STAT pathways interact to control and optimize both innate and adaptive immunity regulations. T cell-specific TSC1-deficient and RICTOR-deficient mice presented increased Tregs number, which was correlated with higher STAT5 activation and proliferation rates (Chen et al., 2013; Park et al., 2013). DCs treated with Torin 1, a catalytic mTOR inhibitor, were found to express lower SOCS3 levels, resulting in increased STAT3 activation, increased B7-H1 expression, and enhanced Tregs induction (Rosborough et al., 2013). In our study, we uncovered that STAT3 was up-regulated in mTOR^KO^ PMN-MDSCs, while its negative regulator Pias was significantly decreased. However, the relationship between mTOR and Pias needs to be further studied. We will carry out further experiments to identify whether STAT3 activation is a direct result of mTOR deficiency or a subsequent signaling cascade.

Adoptive transfusion of MDSCs has been reported to be an effective strategy to control aGVHD after allo-HSCT. *In vitro*-generated MDSCs abrogated allogeneic antigen-specific T cell functions by skewing T cells into Th2, thereby preventing GVHD without affecting the GVL effect (Messmann et al., 2015). In our current study, we found that co-transplanting mTOR^KO^ PMN-MDSCs with grafts could alleviate aGVHD while retaining the GVL effect. In previous studies, adoptive transferred MDSCs were mainly generated *in vitro* by cytokines or isolated from tumor-bearing mice. As BM PMN-MDSCs freshly obtained from WT mice presented limited suppressive function, M-MDSCs with natural powerful suppressive function were considered to be a better source of therapeutic cells (Lin et al., 2018). However, M-MDSCs are present only in a small fraction in the BM. leading to an insufficient amount for treatment. In contrast, PMN-MDSCs possessing a larger proportion and quantity and can be a better choice for cellular immunotherapy. Thus, we assumed that prophylactic infusion of mTOR^KO^ PMN-MDSCs could significantly reduce mortality and alleviate aGVHD. But, it was found before that the intense inflammatory environment could directly undermine the suppressive capacity of MDSCs, which was associated with their conversion toward a mature state (Koehn et al., 2015). Transfusing on day 14 after aGVHD occurrence led to an effect that is less than satisfactory, suggesting that the therapeutic ability of these cells was limited.

In the mTOR^KO^ PMN-MDSC infusion group, the Th1/Th2 ratio was much lower and peripheral proinflammatory cytokines were maintained at a low level in serum, but the Tregs percentage was higher. Combined with the evidence that mTOR^KO^ PMN-MDSCs could only play a preventive role but not a therapeutic effect, we speculated that the adoptive transferred mTOR^KO^ PMN-MDSCs had a suppressive function at the very beginning of aGVHD and further stopped the cytokine storm amplification. Using CD45.1 and CD45.2 to distinguish adoptive transferred and donor-derived PMN-MDSCs, we found that the co-transplanted PMN-MDSCs could only persist for a short time in the BM and PB. Previous studies found that the adoptive transfused MDSCs could be detected in the lymphoid organs and liver until day 30 after transplantation (Messmann et al., 2015). We assumed that co-transplanted MDSCs might rapidly migrate to the peripheral organs or differentiate to a mature state after transfusion. On aGVHD at a later stage, we detected a better quantitative and functional recovery of donor-derived PMN-MDSCs in the mTOR^KO^ PMN-MDSC transfusion group. mTOR^KO^ PMN-MDSC transfusion plays an effective role in aGVHD prevention in a mouse model. However, the application of gene-edited MDSCs in clinical practice needs to be further explored due to the uncertain safety and technical constraints. The mTOR inhibitor rapamycin would become a potential alternative choice to regulate the differentiation and suppressive function of PMN-MDSCs. Our findings provide more evidence for the application of rapamycin for aGVHD control.

Overall, we demonstrated that mTOR is an effective intrinsic regulatory factor for PMN-MDSCs’ differentiation and immunosuppressive function. Also, mTOR^KO^ PMN-MDSC transfusion can be a therapeutic option to prevent cytokine storms at early stages and promote a quantitative and functional recovery of donor-derived PMN-MDSCs in aGVHD. Our findings indicate that mTOR manipulation of PMN-MDSCs might be an effective way in aGVHD prevention.

## Data Availability

The original contributions presented in the study are publicly available. This data can be found here: BioProject: PRJNA726285.
