# Supplementary figures and images for: mTOR Signaling Regulates the Development and Therapeutic Efficacy of PMN-MDSCs in Acute GVHD
Source: Front Cell Dev Biol. 2021 Dec 23;9:741911. doi: 10.3389/fcell.2021.741911 (PMC8733691; doi:10.3389/fcell.2021.741911)

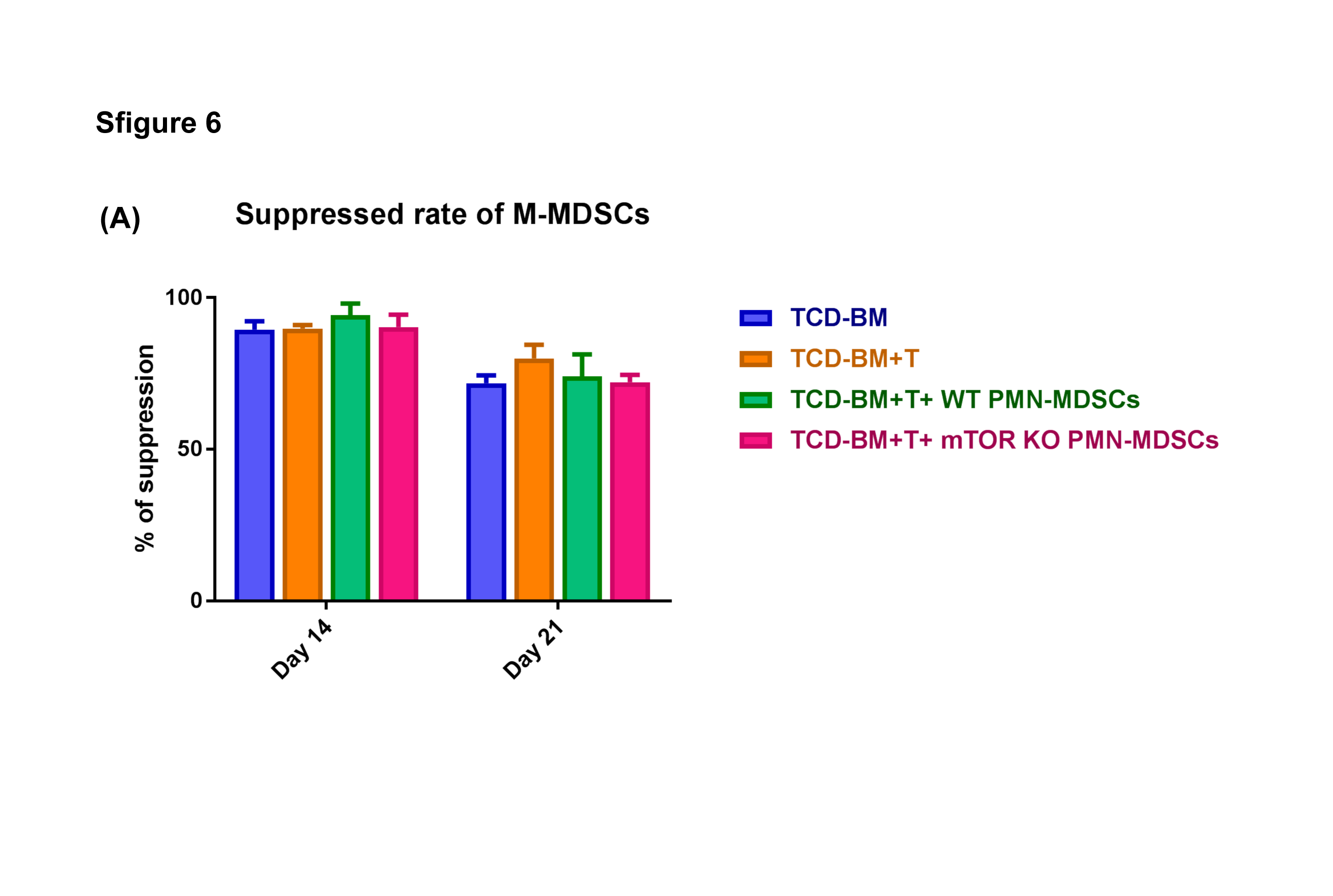

Supplement: Supplementary file 2 [file Image6.TIF]

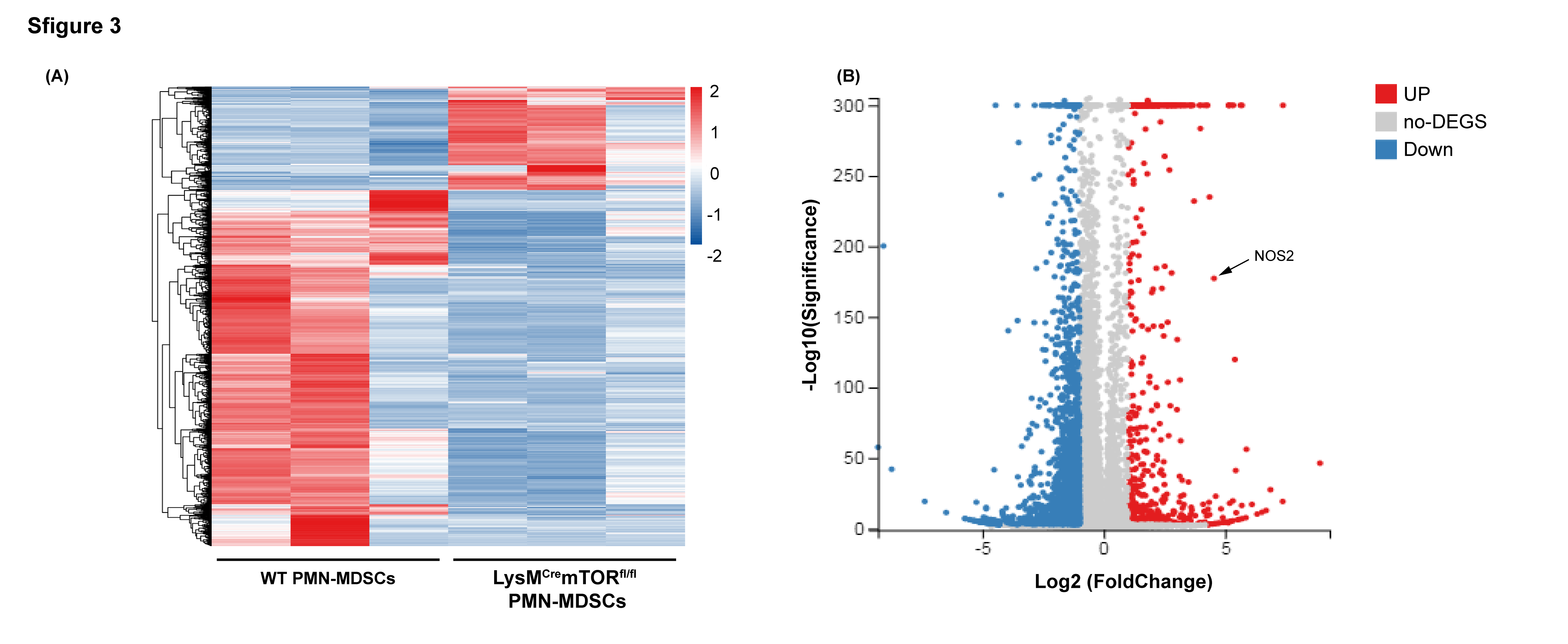

Supplement: Supplementary file 3 [file Image3.TIF]

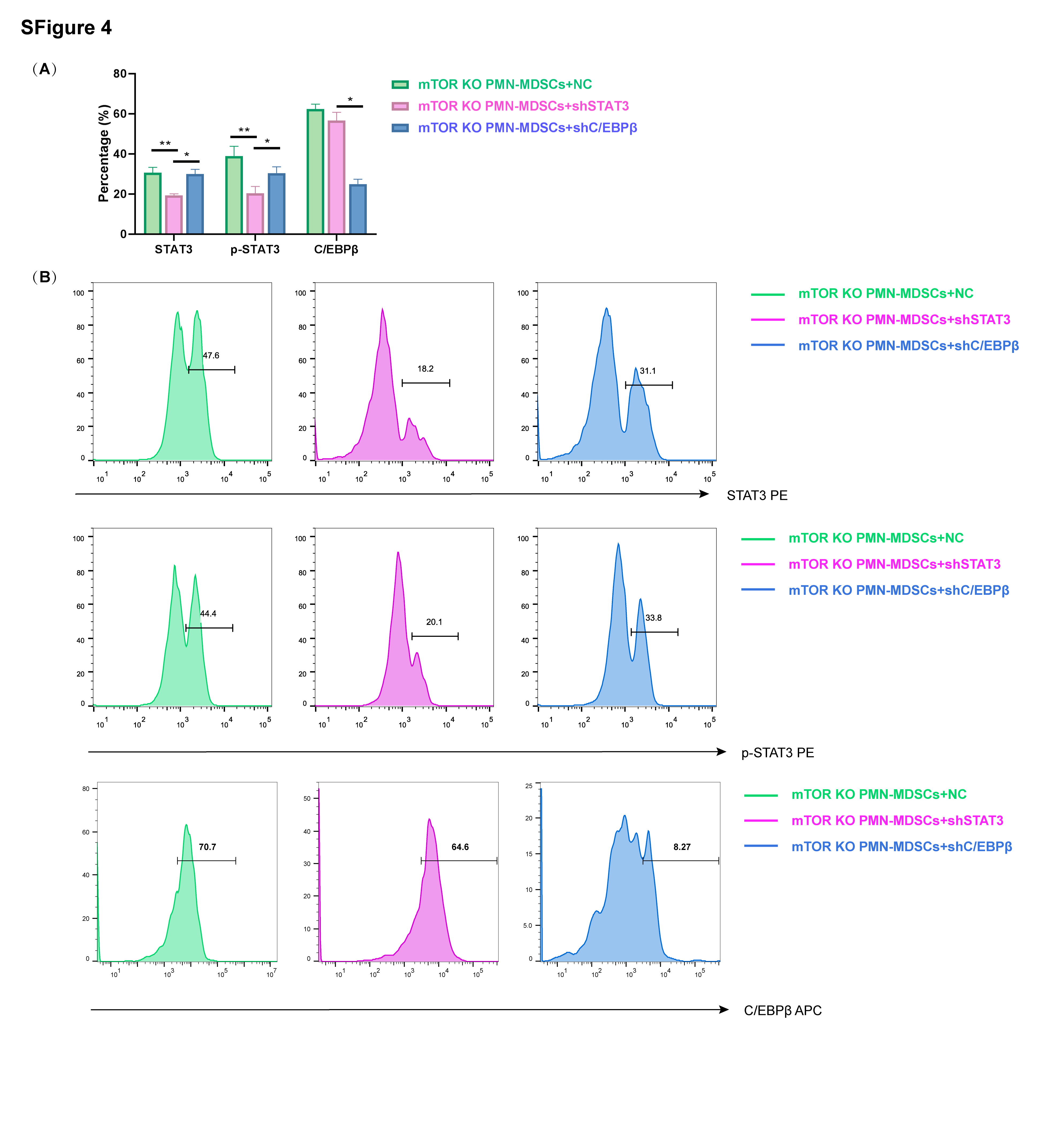

Supplement: Supplementary file 4 [file Image4.TIF]

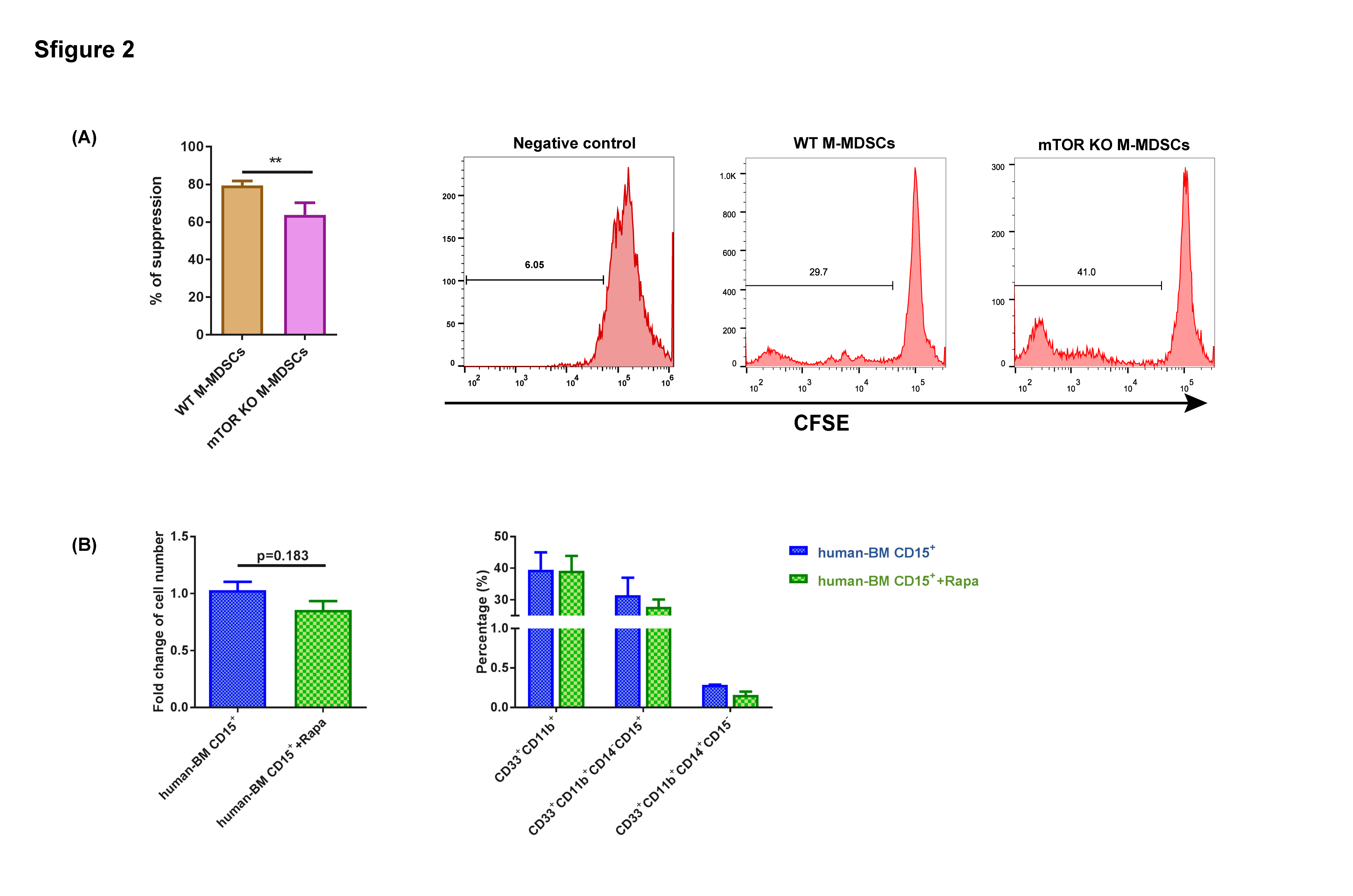

Supplement: Supplementary file 5 [file Image2.TIF]

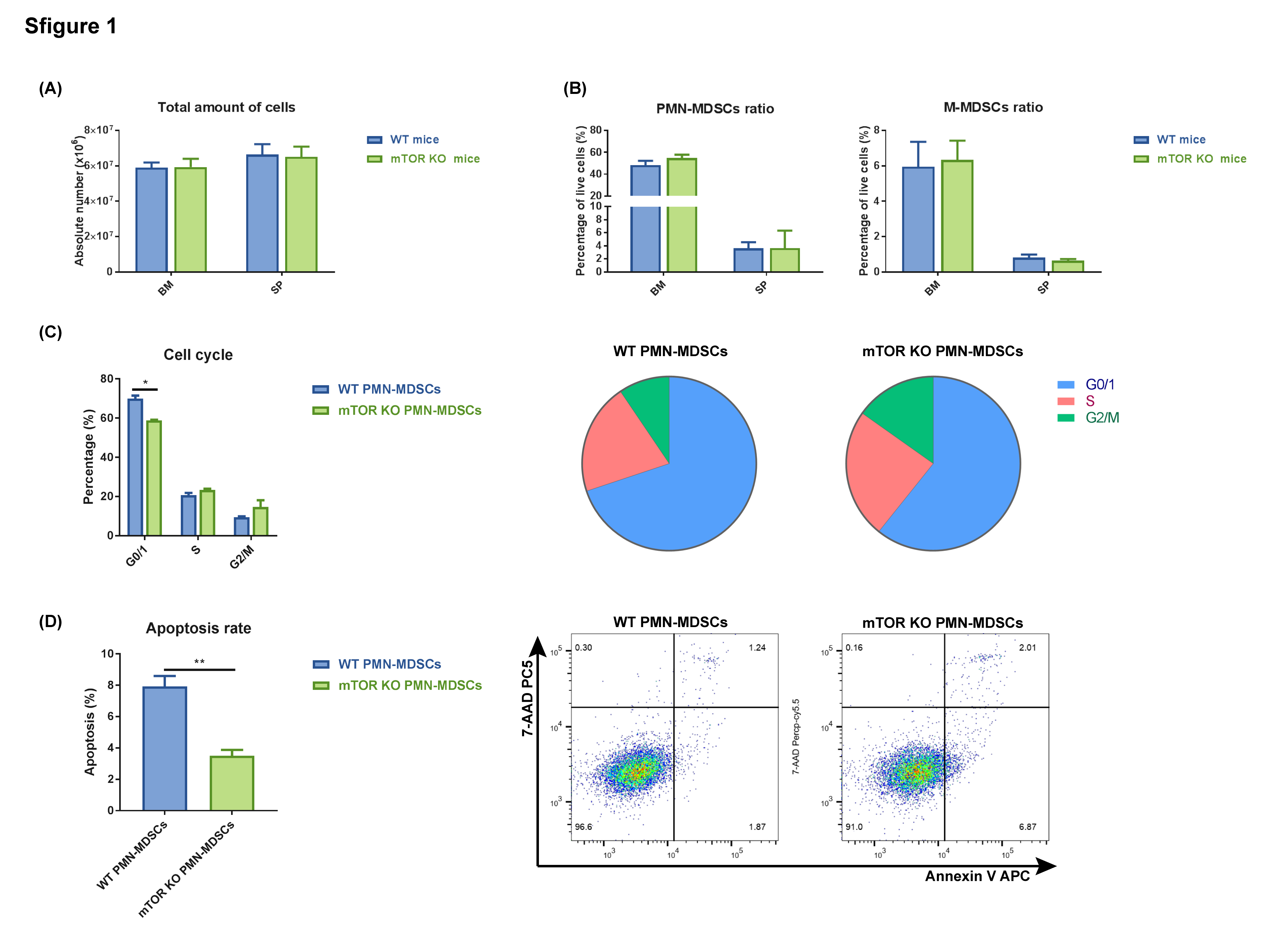

Supplement: Supplementary file 6 [file Image1.TIF]

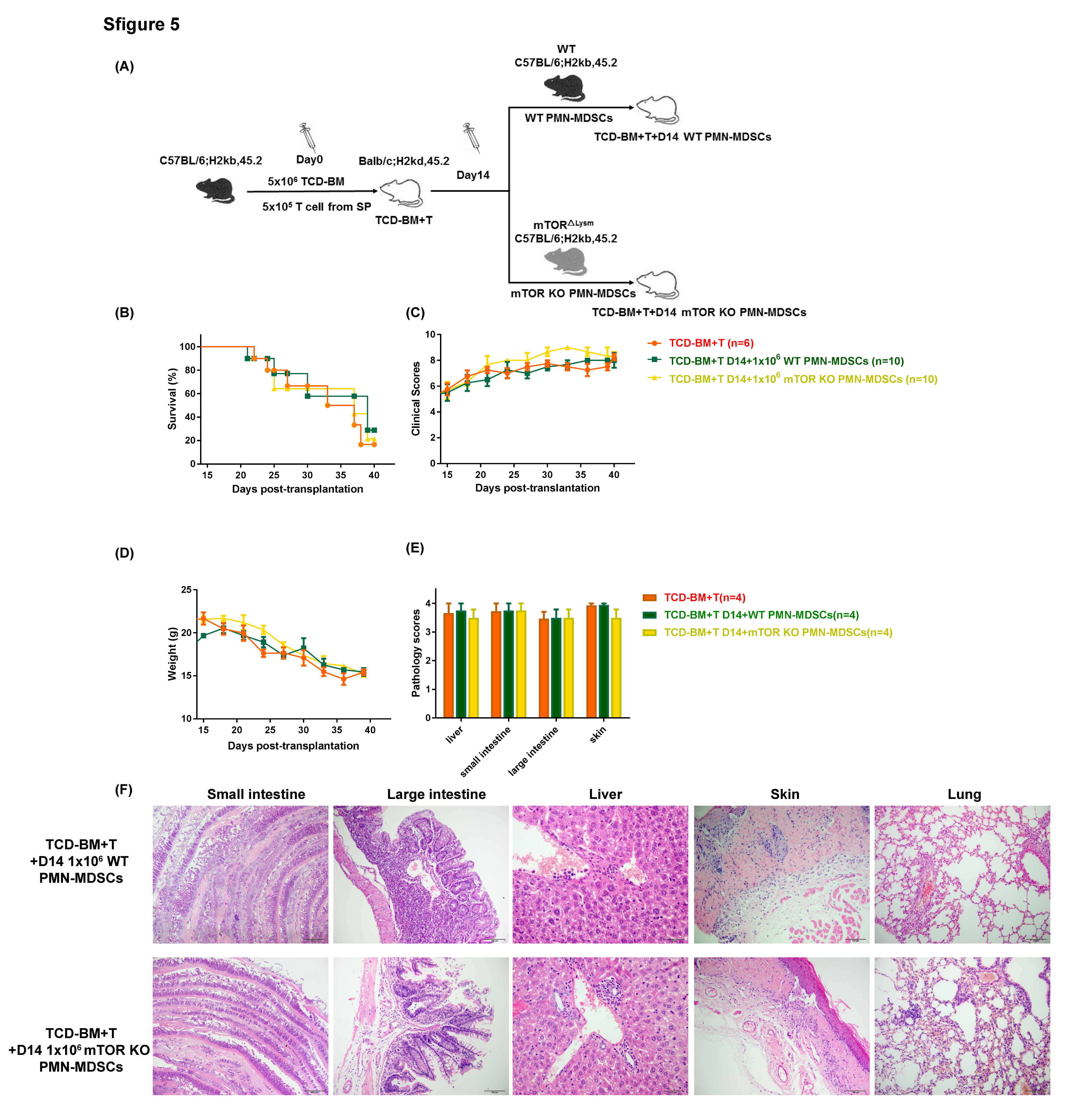

Supplement: Supplementary file 8 [file Image5.TIF]
