# Supplementary material for: mTOR Signaling Regulates the Development and Therapeutic Efficacy of PMN-MDSCs in Acute GVHD
Source: Front Cell Dev Biol. 2021 Dec 23;9:741911. doi: 10.3389/fcell.2021.741911 (PMC8733691; doi:10.3389/fcell.2021.741911)
Supplement: Supplementary file 9 [file DataSheet1.docx]

**Description of Supplementary Files**

**Supplementary Figure Legends**

**Supplementary figure 1. The effect of conditional knockout** **mTOR on physical and functional characteristics of PMN-MDSCs**

(A-B) No significant statistical variations were observed in total BM cells (A) and proportions of PMN-MDSCs and M-MDCSs from BM and SP (B) between mTOR^fl/fl^ and LysM-Cre mTOR^fl/fl^ mice. (C-D) PMN-MDSCs were isolated from mTOR^fl/fl^ and LysM-Cre mTOR^fl/fl^ mice and assayed for cell cycle (C) and cell viability (D) after co-culturing with active T cells for 3 days. Statistical comparison was performed using MWU test, * *p* < 0.05, ** *p* < 0.01.

**Supplementary figure 2. mTOR signal affected the functional characteristics of M-MDSCs and human CD15+ bone marrow cells.**

(A) BM-derived WT or mTOR^KO^ M-MDSCs were mixed with CFSE-labeled splenic T cell at a ratio of 1:1 for 3.5 days. The suppression of splenic T cells proliferation was analyzed and calculated as the suppressive ability of M-MDSCs (mean ± SD; n=8). (B) CD15^+^ BM cells isolated from healthy donors were pretreated RAPA for 4 hours. RAPA treatment did not affect the cell viability or influence cell differentiation for a short duration (mean ± SD; n=6). Experiments were repeated 2-3 times with at least 3 mice per group and 3 healthy donors with similar results. Statistical comparison was performed using the two-tailed unpaired Student *t*-test or MWU test, * *p* < 0.05, ** *p* < 0.01.

**Supplementary figure 3. Gene expression in PMN-MDSCs from** **mTOR^fl/fl^ and LysM-Cre mTOR^fl/fl^.**

Whole transcriptome of PMN-MDSCs differentiated in mTOR^fl/fl^ (WT) versus LysM-Cre mTOR^fl/fl^ (mTOR^KO^) PMN-MDSCs (A) Heatmap showed differential expression of genes between WT and mTOR^KO^ PMN-MDSCs. (B) Colors indicate significant upregulation (at least 1.5-fold red) or downregulation (at least 1.5-fold blue).

**Supplementary figure 4. Validation of STAT3 shRNA knockdown efficiency.**

(A) The transfection efficiencies for shSTAT3 and shC/EBPβ were verified by flow cytometry analysis of labeling STAT3, p-STAT3 and shC/EBPβ (mean ± SD; n=5). MWU test was performed to assess significance (* *p* < 0.05, ** *p* < 0.01). (B) Representative flow-cytometric histograms of different groups were shown.

**Supplementary figure 5. The therapeutic effect was diminished when transfusing** **mTOR^KO^ PMN-MDSCs after the occurrence of aGVHD**

BALB/c recipients received WT or mTOR^KO^ PMN-MDSCs day 14 post-transplantation after aGVHD onset. (A) Schematic diagram of experiment procedures. (B-E) All of the aGVHD indicators, including survival time (B), aGVHD clinical scores (C), weight changes (D) and histopathological score (E) displayed no significant difference between two transfusion groups. (F) Representative section of targeted organs were shown in each groups. The data were presented as mean ± SEM, which combined from two independent experiments with at least 6 mice per group. Numerals in brackets indicated the number of mice tested. Survival curves were compared using the log-rank test, and the other aGVHD indicators were assessed by MWU test.

**Supplementary figure 6. Suppressive function of M-MDSCs in mTOR^KO^ PMN-MDSCs transfused aGVHD mice.**

(A) M-MDSCs were isolated from BM in each groups on day 14 and 21, and co-cultured with active SP cells to detect the suppressive ability. Data represented the mean± SD of one representative experiment out of three experiments performed. Unpaired two-tailed Student *t*-test was performed to assess significance (* *p* < 0.05, ** *p* < 0.01)

**Supplementary figure 7. Neutrophil development in mTOR^fl/fl^ and LysM-Cre mTOR^fl/fl^ mice**

(A) The gating strategy of pre-neutrophils, immature neutrophils and mature neutrophils in BM from mTOR^fl/fl^ and LysM-Cre mTOR^fl/fl^ mice (left). The percentage of three stages of neutrophils out of Gr1^+^CD11b^+^ cells in mTOR^fl/fl^ and LysM-Cre mTOR^fl/fl^ mice (right).

**Supplementary Table Legends**

**Supplementary Table1. Antibodies for flow cytometry analysis and Western blot**

**Supplementary Table2. Primers used for qPCR**
